# Retrospective evaluation of the role of gemcitabine‐docetaxel in well‐differentiated and dedifferentiated liposarcoma

**DOI:** 10.1002/cam4.5298

**Published:** 2022-09-24

**Authors:** Prapassorn Thirasastr, Heather Lin, Behrang Amini, Wei‐Lien Wang, Jeffrey M. Cloutier, Elise F. Nassif, Emily Z. Keung, Christina L. Roland, Barry Feig, Dejka Araujo, Robert S. Benjamin, Anthony P. Conley, John A. Livingston, Joseph Ludwig, Shreyaskumar Patel, Ravin Ratan, Vinod Ravi, Maria Alejandra Zarzour, Xiao Zhou, Neeta Somaiah

**Affiliations:** ^1^ Department of Sarcoma Medical Oncology The University of Texas MD Anderson Cancer Center Houston Texas USA; ^2^ Department of Biostatistics The University of Texas MD Anderson Cancer Center Houston Texas USA; ^3^ Department of Musculoskeletal Imaging, Division of Diagnostic Imaging The University of Texas MD Anderson Cancer Center Houston Texas USA; ^4^ Department of Pathology, Division of Pathology/Laboratory Medicine The University of Texas MD Anderson Cancer Center Houston Texas USA; ^5^ Department of Surgical Oncology, Division of Surgery The University of Texas MD Anderson Cancer Center Houston Texas USA

## Abstract

**Objective:**

Well‐differentiated (WDLPS) and dedifferentiated liposarcoma (DDLPS) account for the majority of liposarcomas. Although gemcitabine‐docetaxel is used as second‐line treatment in soft tissue sarcomas, its efficacy in WDLPS/DDLPS is not established. This study retrospectively analyzed the efficacy of gemcitabine regimens in WDLPS/DDLPS.

**Methods:**

All patients with WDLPS or DDLPS who received gemcitabine‐based chemotherapy at our institution between September 2002 and January 2021 were included. Response was evaluated by an independent radiologist using RECIST 1.1. The Kaplan–Meier method was used to estimate distributions of survival outcomes and log‐rank tests were used to compare survival outcomes between subgroups.

**Results:**

Sixty‐five WDLPS/DDLPS patients were included. Seven patients (10.8%) received a gemcitabine‐based regimen more than once, totaling 72 treatments. The median age at the start of treatment was 66 years (range 32–80 years). Sixty‐five (90.3%) regimens were gemcitabine‐docetaxel, and 7 (9.7%) were gemcitabine alone. Majorities of treatments were for disease that was recurrent/metastatic (86.1%), was abdominal/retroperitoneal (83.3%), and had DDLPS components (88.9%), while 25.0% of treatments were for multifocal disease. The overall response rate was 9.7% (7/72). All responses were in patients with documented DDLPS. The median time to progression was 9.2 months (95% CI 5.3–12.3 months). The median overall survival from the start of therapy was 18.8 months (95% CI 13.1–32.4 months).

**Conclusion:**

Gemcitabine‐docetaxel is an efficacious second‐line treatment for DDLPS. Though cross study comparisons are not advisable, response to gemcitabine‐docetaxel compares favorably to current standard options trabectedin and eribulin. This combination is a valid comparator arm for future second‐line trials in DDLPS.

## 1 INTRODUCTION

Liposarcoma (LPS) is one of the most common types of soft tissue sarcoma (STS).^1^ Well‐differentiated LPS (WDLPS) and dedifferentiated LPS (DDLPS) are the major subtypes, accounting for around 60% of malignant adipocytic tumors. WDLPS and DDLPS encompass a spectrum of low‐ to high‐grade tumors that share the same genetic alteration on chromosome 12q13‐15, resulting in overexpression of oncogenes including *MDM2* (mouse double minute 2), *CDK4* (cyclin‐dependent kinase 4), and HMGA2 (high mobility group AT‐hook 2).^2^, ^3^ Local and often multifocal recurrence is common following resection of both WDLPS and DDLPS; however, the clinical course and prognosis of the 2 subtypes are distinct. A pure WDLPS, defined based on the absence of DDLPS in the tumor, has very low metastatic potential and is not responsive to cytotoxic chemotherapy, while the presence of DDLPS is associated with shorter time to recurrence and a high metastatic potential with as much as a 10%–25% rate of metastasis in 3 years.^4^, ^5^ Therefore, a heterogenous LPS with the presence of confirmed dedifferentiation is classified as DDLPS. Although surgery remains the mainstay of treatment for both WDLPS and DDLPS when feasible, systemic treatments are essential in the setting of locally advanced/unresectable, multiply recurrent, or metastatic disease, as is often encountered in the case of DDLPS.

Owing to the rarity of the disease, and prior trials not evaluating subtype‐specific efficacy, the data for chemotherapy efficacy are limited so far. Commonly used chemotherapies include regimens standard for STS, such as doxorubicin combinations, gemcitabine‐docetaxel, and more recently trabectedin and eribulin. Given the presence of MDM2 and CDK4 amplification in WDLPS/DDLPS cases, both MDM2 inhibitors and CDK4/6 inhibitors are being actively evaluated in this subtype, with ongoing phase 2/3 trials. CDK4/6 inhibitor studies are further along; palbociclib, ribociclib, and abemaciclib, have shown activity in phase 2 LPS trials.^6^, ^7^, ^8^

Data from 2 retrospective studies have shown that the response rate (RR) of doxorubicin‐based combination chemotherapy for LPS ranged from 12% to 21% and the median progression‐free survival (PFS) ranged from 4 to 4.6 months in the first‐line setting. Despite the frequent use of gemcitabine‐docetaxel as second‐line treatment for STS, the efficacy of the gemcitabine‐docetaxel regimen in the WDLPS and DDLPS subtypes is not well established. In large randomized studies of gemcitabine‐docetaxel in STS, only a relatively small number of high‐grade LPS patients were included (16.4%^9^ and 3.9%^10^), leading to questions about the true applicability of the data to these subtypes. This retrospective analysis aims to define the efficacy of gemcitabine‐docetaxel in DDLPS by analyzing our experience over the past 20 years at The University of Texas MD Anderson Cancer Center.

## 2 MATERIALS AND METHODS

### 2.1 Patient selection

Patients with pathologically confirmed WDLPS or DDLPS treated with gemcitabine‐based regimens were identified using electronic medical records (Epic SlicerDicer search: January 2014 to January 2021; tumor registry search: September 2002 to March 2014). Inclusion criteria included age ≥ 18 years, treatment with at least 1 cycle of gemcitabine‐based chemotherapy, and follow‐up imaging available in our system. The diagnosis was established according to the 2013 World Health Organization Classification of Tumors by expert pathologists.^11^

This study protocol was approved by the Institutional Review Board of MD Anderson Cancer Center (Protocol 2021–1346).

### 2.2 Data collection

Patients' data were obtained from electronic medical records and the tumor registry database of MD Anderson. All patients that met our inclusion criteria had their data entered into a database created for this protocol in the MD Anderson domain of Research Electronic Data Capture (REDCap). Baseline characteristics, pathology details, molecular tests, and details of the treatment received were collected. If the chemotherapy regimen was rechallenged without intervening definitive surgery, only the first record was included. Treatment de‐escalation from gemcitabine‐docetaxel to gemcitabine as single agent in the same recurrence was also excluded. Multiple records of gemcitabine‐based regimens would only be documented when used to treat different recurrences, meaning the patient receiving intervening surgery. This non‐interventional study was exempt from an informed consent requirement.

### 2.3 Treatment

All patients were treated according to their primary medical oncologist's decision based on standard treatment (gemcitabine or gemcitabine‐docetaxel) and relevant clinical studies (gemcitabine‐docetaxel‐olaratumab) available at that time. The cases were often managed by a multidisciplinary team with a surgeon on board for any local treatment interventions when appropriate.

### 2.4 Efficacy assessment

Treatment response was evaluated by contrast‐enhanced computerized tomography (CT) scans and occasionally by positron emission tomography (PET)/CT scans every 2–3 cycles a in accordance with standard practice. PET/CT scans have been shown to be valuable in identifying dedifferentiation more accurately in heterogeneous tumors compared to evaluation based on characteristics in CT scan alone and helpful in evaluating response in the DDLPS component.^12^, ^13^ The best response to treatment, imaging at the time of best response, and imaging at the time of progression of disease were reviewed by an independent board‐certified radiologist with specialized expertise in sarcomas using Response Evaluation Criteria in Solid Tumors (RECIST) 1.1 criteria for the purpose of this study.

Additional efficacy outcomes were also evaluated: time to progression (TTP), PFS, and overall survival (OS). In this study, as response was determined by treating physicians at the time of treatment on the basis of their assessment of the images and the radiologic report, we recorded both progression as defined by the treating physician and as defined by an independent radiologist using RECIST 1.1. Surgical specimens from tumor resection after treatment with gemcitabine‐based therapy were also reviewed by an independent sarcoma pathologist.

### 2.5 Statistical analyses

Descriptive statistics (frequency distribution, mean [±standard deviation], and median [range]) were used to summarize patients' baseline characteristics. Efficacy analyses of gemcitabine‐based chemotherapy including RR, TTP, and PFS were performed only in the locally advanced, recurrent, or metastatic setting, when measurable disease was present. The rate of RECIST 1.1 response was assessed by an independent radiologist as described previously. The Kaplan–Meier method^14^ was used to estimate TTP, PFS and OS. TTP was defined as the time from initiation of each treatment to disease progression. The use of gemcitabine‐based chemotherapy more than once for different recurrences in the same patient were assessed as independent instances for purposes of RR and TTP. This occurred when patients underwent resection for localized or locally advanced disease at some point after systemic therapy and went on to receive another line of the therapy once the disease recurred. PFS was defined as the time from the last gemcitabine‐based treatment initiation (for the most recent recurrence) to the time of progression or death, whichever occurred first. OS was analyzed as (1) the time from initial diagnosis to death from any cause and (2) the time from initiation of the last instance of gemcitabine‐based chemotherapy to death. For events that had not occurred by the time of the data cut‐off, times were censored at the last contact at which the patient was known to be progression free or dead for TTP and PFS or at the last time the patient was known to be alive for OS. The log‐rank test was performed to test the difference in survival between groups.^15^ Regression analyses of survival data based on the Cox proportional hazards (PH) model^16^ were conducted on TTP and OS in univariate and multivariate setting. For the analysis of TTP in relation to best response, the landmark analysis method was used and date of best response evaluation was considered the starting point of TTP. Statistical significance was determined using a 2‐sided *p*‐value <0.05. All analyses were conducted using SAS (version 9.4), S‐PLUS (version 8.04, TIBCO Software Inc.) and R (version 3.5.3 [2019‐03‐11]).

## 3 RESULTS

### 3.1 Patients and treatments

**FIGURE 1:**
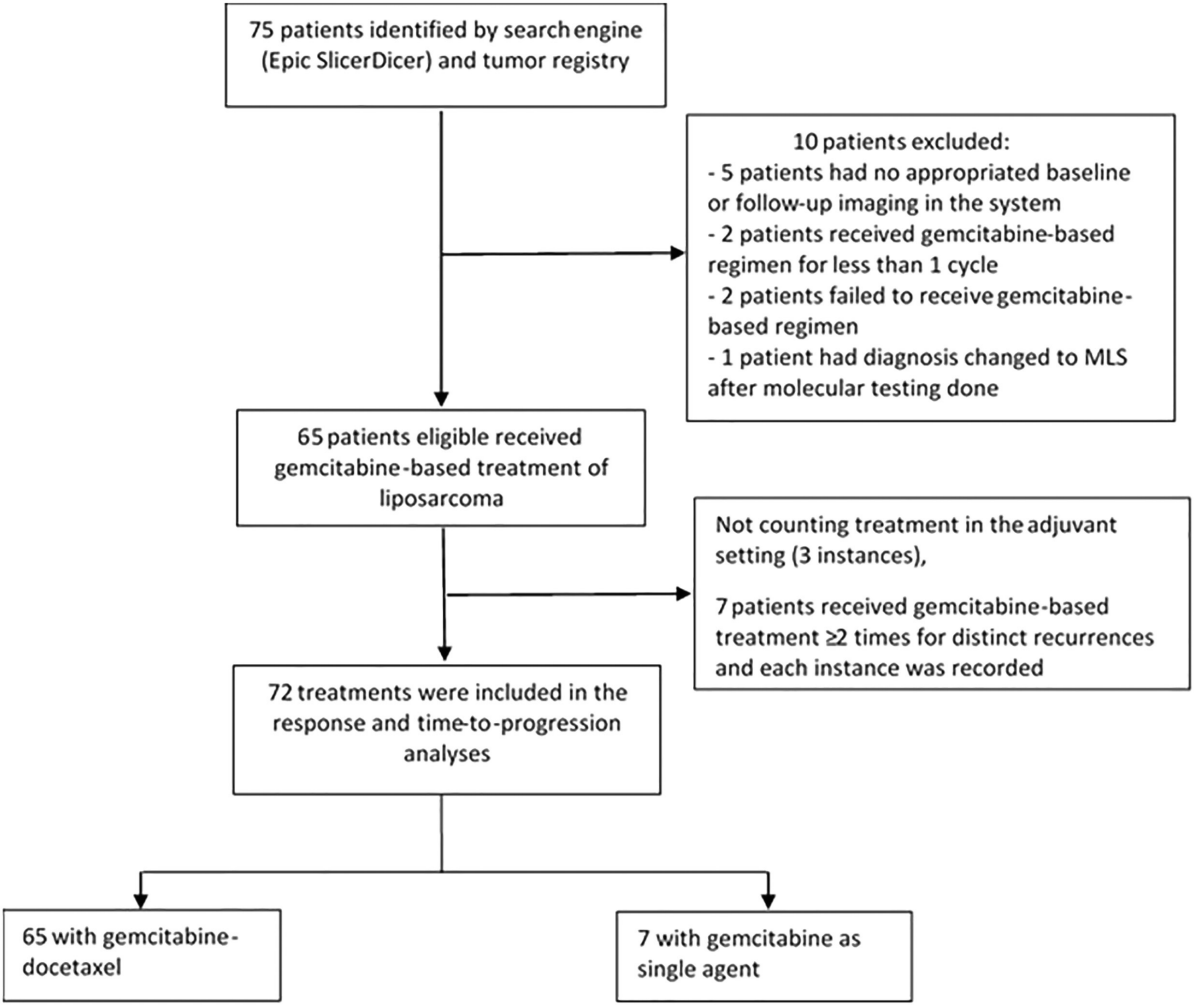
Flow chart of patients who met inclusion/exclusion criteria for the study population.

We identified 75 patients with pathologically confirmed WDLPS and DDLPS who received gemcitabine and docetaxel or single‐agent gemcitabine from September 2002 to January 2021 (Figure 1). Of the 75 patients, 65 patients met our predefined eligibility criteria for inclusion, and among those 65 patients, 7 patients (10.8%) received gemcitabine‐based therapy more than once for different recurrences with intervening surgical resection, totaling 72 instances of gemcitabine‐based chemotherapy.

Of the 72 total treatments, 65 (90.3%) were gemcitabine‐docetaxel therapy and 7 (9.7%) were single‐agent gemcitabine (Table 2). Gemcitabine‐based therapy was most often used in the second line (56.9%) and was used in the first line in 19.4% of treatments. A median of 4 cycles (range 1–25 cycles) were received, with a median treatment duration of 2.9 months (range 0.4–19.5 months). The major reason for discontinuation was disease progression as determined by the treating physician (33 instances, 45.8%), followed by discontinuation due to initiation of local therapy (16 instances, 22.2%) and toxicity (15 instances, 20.8%). Other reasons for stopping treatment included patients' decision for a treatment break (3 instances, 4.2%), ruptured brain aneurysm associated with neither the treatment nor the malignancy (1 instance, 1.4%), interruption for obtaining an additional tissue diagnosis for a secondary malignancy (1 instance, 1.4%), completion of the planned number of cycles (1 instance, 1.4%), and sudden death due to an unknown cause (1 instance, 1.4%).

At the time of gemcitabine‐based treatment initiation, of the 65 patients included, the median age was 66 years (range 32–80 years, Table 1). The majority of the patients (41 patients, 63.1%) were white (Table 1). A secondary malignancy was present in 17 patients (26.2%), of which prostate cancer was the most common (5 patients, 29.4%). The retroperitoneum was the predominant primary site (40 patients, 61.5%). Less frequent primary sites were extremities and the inguinal canal, accounting for 5 patients each (7.7%). Forty‐eight patients (73.9%) were diagnosed with DDLPS at baseline. At the initiation of gemcitabine‐based treatment, 57 patients (87.7%) had a DDLPS component, all 7 patients who had gemcitabine‐based treatment more than once were in the DDLPS group leading to a total of 64 instances (64/72, 88.9%) with DDLPS component.

**TABLE 1:** Baseline characteristics

Characteristic		Frequency *N* = 65	Percentage (%)
Age at treatment, years (median, range)		66 (32–80)	
Sex	Female	28	43.08
	Male	37	56.92
Race	White	41	63.08
	Asian	9	13.85
	African American	3	4.62
	Other	9	13.85
	Missing or unknown	3	4.62
Ethnicity	Hispanic or Latino	14	21.54
	Not Hispanic or Latino	47	72.31
	Missing or unknown	4	6.16
Presence of secondary malignancy	Yes^a^	17	26.15
	No	48	73.85
Histologic subtype at diagnosis	WDLPS	17	26.15
	DDLPS^b^	48	73.85
Histologic subtype in overall clinical course	WDLPS	4	6.15
	DDLPS^b^	61	93.85
Primary tumor location	Retroperitoneum	40	61.54
	Inguinal canal	5	7.69
	Extremities	5	7.69
	Others^c^	15	23.08
Stage at diagnosis	Localized	41	63.08
	Localized with multifocal	16	24.62
	Metastatic	8	12.31
Primary treatment	Surgery	62	95.38
	Systemic treatment	24	36.92
	Radiation	11	16.92
Total number of systemic treatments received	1–2 lines	22	33.85
	3–4 lines	32	49.23
	>4 lines	11	16.92

Abbreviations: DDLPS, dedifferentiated liposarcoma; DLPS, well‐differentiated liposarcoma.

^a^ Secondary malignancy includes prostate cancer in 5 patients (29.41%), malignant melanoma in 2 patients (11.76%), bladder cancer in 2 patients (11.76%), leukemia in 2 patients (11.76%), renal cell carcinoma in 1 patient (5.88%), breast cancer in 1 patient (5.88%), ductal carcinoma in situ of breast in 1 patient (5.88%), colon cancer in 1 patient (5.88%), thyroid cancer in 1 patient (5.88%), and skin cancer in 1 patient (5.88%).

^b^ Classified with DDLPS if any dedifferentiated component was present.

^c^ Others include intraabdominal in 6 patients (9.23%), superficial trunk in 1 patient (1.54%), chest in 1 patient (1.54%), bladder in 1 patient (1.54%), small bowel in 1 patient (1.54%), colon in 1 patient (1.54%), mesentery in 1 patient (1.54%), pancreatic tail in 1 patient (1.54%), pelvic cavity in 1 patient (1.54%), and presacral space in 1 patient (1.54%).

Ten instances (13.9%) of gemcitabine‐based treatment were given in a primary, localized/multifocal disease setting, and all were neoadjuvant therapy (Table 2). Among these 10 treatments, most were for disease that was localized (9/10, 90%), was in the abdomen/retroperitoneum (9/10, 90%), and had DDLPS present (9/10, 90%). Only 1 of these patients had multifocal disease before the treatment. Only 2 of these patients received the gemcitabine‐based treatment as first‐line systemic treatment, while the remaining 8 patients received it as second‐line treatment. Nine of the treatments (90%) were gemcitabine‐docetaxel and 1 was gemcitabine alone. A median of 3 cycles were received (range 1–6 cycles). Six patients (60%) stopped as planned for local treatment, 2 stopped due to toxicity, and the remaining 2 patients stopped due to disease progression by physician assessment, but none had met the RECIST 1.1 criteria for disease progression by radiologist review. In all 10 instances, the patients received surgery after the gemcitabine‐based treatment: immediately after the treatment in 6 instances, and after further treatment with other chemotherapy and/or radiation in 4 instances.

**TABLE 2:** Characteristics of gemcitabine‐based therapy and details of treatment in all instances (*n* = 72) and by setting

Parameter No., (%)	Total (*n* = 72)	Setting		
		Localized/multifocal (*n* = 41)		Metastatic (*n* = 31)^a^
		Primary (*n* = 10)^b^	Recurrent (*n* = 31)^b^	
Gemcitabine‐based regimen				
Gemcitabine‐docetaxel	65 (90.28)	9 (90.0)	29 (93.55)	27 (87.10)
Gemcitabine single agent	7 (9.72)	1 (10.0)	2 (6.45)	4 (12.90)
Subtype				
DDLPS	64 (88.89)	9 (90.0)	26 (83.87)	29 (93.55)
WDLPS	8 (11.11)	1 (10.0)	5 (16.13)	2 (6.45)
Line of gemcitabine‐based treatment				
First line	14 (19.44)	2 (20.0)	8 (25.81)	4 (12.90)
Second line	41 (56.94)	8 (80.0)	15 (48.39)	18 (58.06)
Third line or later	17 (23.61)	0 (0)	8 (25.81)	9 (29.03)
Number of cycles received				
<4	32 (44.44)	6 (60.0)	15 (48.39)	11 (35.48)
≥4 cycles	39 (54.17)	4 (40.0)	15 (48.39)	20 (64.52)
Missing data	1 (1.39)	0	1 (3.23)	0
Reason for discontinuation				
Disease progression determined by treating physician	23 (31.95)	2 (20.0)	10 (32.26)	17 (54.84)
Confirmed by RECIST 1.1	10 (13.89)	0	4 (12.90)	6 (19.35)
Planned local treatment	16 (22.22)	6 (60.0)	7 (22.58)	3 (9.68)
Toxicity	15 (20.83)	2 (20.0)	5 (16.13)	8 (25.81)
Gemcitabine‐docetaxel (*n* = 65)	14 (21.54)	2	5	7
Gemcitabine (*n* = 7)	1 (14.29)	0	0	1
Other^c^	7 (9.72)	0	5 (16.13)	2 (6.45)
Surgery after gemcitabine‐based therapy				
Yes	32 (44.44)	10 (100.0)	18 (58.06)	4 (12.90)
Right after	21 (29.17)	6 (60.0)	12 (38.71)	3 (9.68)
Following other intervening therapy (radiation/other chemotherapy)	11 (15.28)	4 (40.0)	6 (19.35)	1 (3.23)
No	40 (55.56)	0	13 (41.94)	27 (87.10)
Pathologic response for patients undergoing surgery (*n* = 19)^d^				
Presence of tumor necrosis				
≥30%	4 (21.05)	3 (60.0)	1 (9.09)	0
5% to <30%	7 (36.84)	1 (20.0)	5 (45.45)	1 (33.33)
No tumor necrosis	8 (42.11)	1 (20.0)	5 (45.45)	2 (66.67)
Presence of tumor hyalinization as treatment effect (*n* = 18)				
≥30%	6 (33.33)	1 (25.0)	4 (36.36)	1 (33.33)
>12.5% but <30%	4 (22.22)	2 (50.0)	2 (18.18)	1 (33.33)
≤12.5%^e^	8 (44.44)	1 (25.0)	5 (45.45)	1 (33.33)

Abbreviations: WDLPS, well‐differentiated liposarcoma; DDLPS, dedifferentiated liposarcoma.

^a^ Metastatic sites included peritoneum (26/31, 83.87%), subcutaneous/intramuscular tissue (6/31, 19.35%), lung (5/31, 16.13%), liver (5/31, 16.13%), bone (5/31, 16.13%), lymph node (3/31, 9.68%), brain (1/31, 3.23%), adrenal gland (1/31, 3.23%), and colon (1/31, 3.23%).

^b^ Among patients in primary and recurrent settings, 1 (10.0%) and 17 (54.84%) patients, respectively, had multifocal disease.

^c^ Other reasons for treatment discontinuation included 3 treatment breaks (4.17%), 1 ruptured brain aneurysm (1.39%), 1 to evaluate possible secondary malignancy (1.39%), 1 completed treatment (1.39%), and 1 death (1.39%).

^d^ 19 of 21 cases with available tumor specimens underwent surgery right after gemcitabine‐based treatment.

^e^ A hyalinization cut‐point at >12.5% was used based on improvement of relapse‐free survival and OS in patients with >12.5% hyalinization in surgical specimens.^27^

Thirty‐one instances (43.1%) of gemcitabine‐based treatment were given in a recurrent, localized/multifocal setting. Of these treatments, 17 (54.8%) were for multifocal disease, 27 (87.1%) were for disease in the abdomen/retroperitoneum, and 26 (83.9%) were for disease with a DDLPS component. Most of these treatments (29/31, 93.6%) were gemcitabine‐docetaxel while 2 (6.5%) were gemcitabine alone. Fourteen treatments (14/31, 45.2%) were stopped due to progression by physician assessment, and among these cases, 4 (4/31, 12.9%) were confirmed as progression by RECIST 1.1. Other treatments were stopped due to toxicity in 5 instances (16.1%), local therapy in 7 instances (22.6%), a treatment break in 3 instances (9.7%), death from an unknown cause in 1 instance (3.2%), and completion of planned cycles in 1 instance (3.2%). In this setting, 18 instances (58.1%) of the gemcitabine‐based treatment were followed by surgery: immediately after the treatment in 12 instances (38.7%), and after additional regimens/radiation in 6 (19.4%).

Another 31 instances (43.1%) of gemcitabine‐based treatment were given as palliative treatment in a metastatic setting. Of these treatments, 24 (77.4%) were for disease in the abdomen/retroperitoneum and 29 (93.6%) were for disease with a DDLPS component. Twenty‐seven treatments (87.1%) were gemcitabine‐docetaxel while 4 (12.9%) were gemcitabine alone. Seventeen treatments (17/31, 54.8%) were stopped due to progression of disease, of which 6 (6/31, 19.4%) were confirmed as progression by RECIST 1.1. Other reasons for stopping treatment included toxicity in 8 instances (25.8%), local therapy in 3 (9.7%), ruptured brain aneurysm in 1 (3.2%) and patient's preference for a treatment break in 1 (3.2%). In this palliative group, 4 treatments (12.9%) were followed by surgeries, of which 3 (9.7%) were immediately after the treatment and 1 (3.2%) was after other subsequent treatment.

### 3.2 Efficacy

**FIGURE 2:**
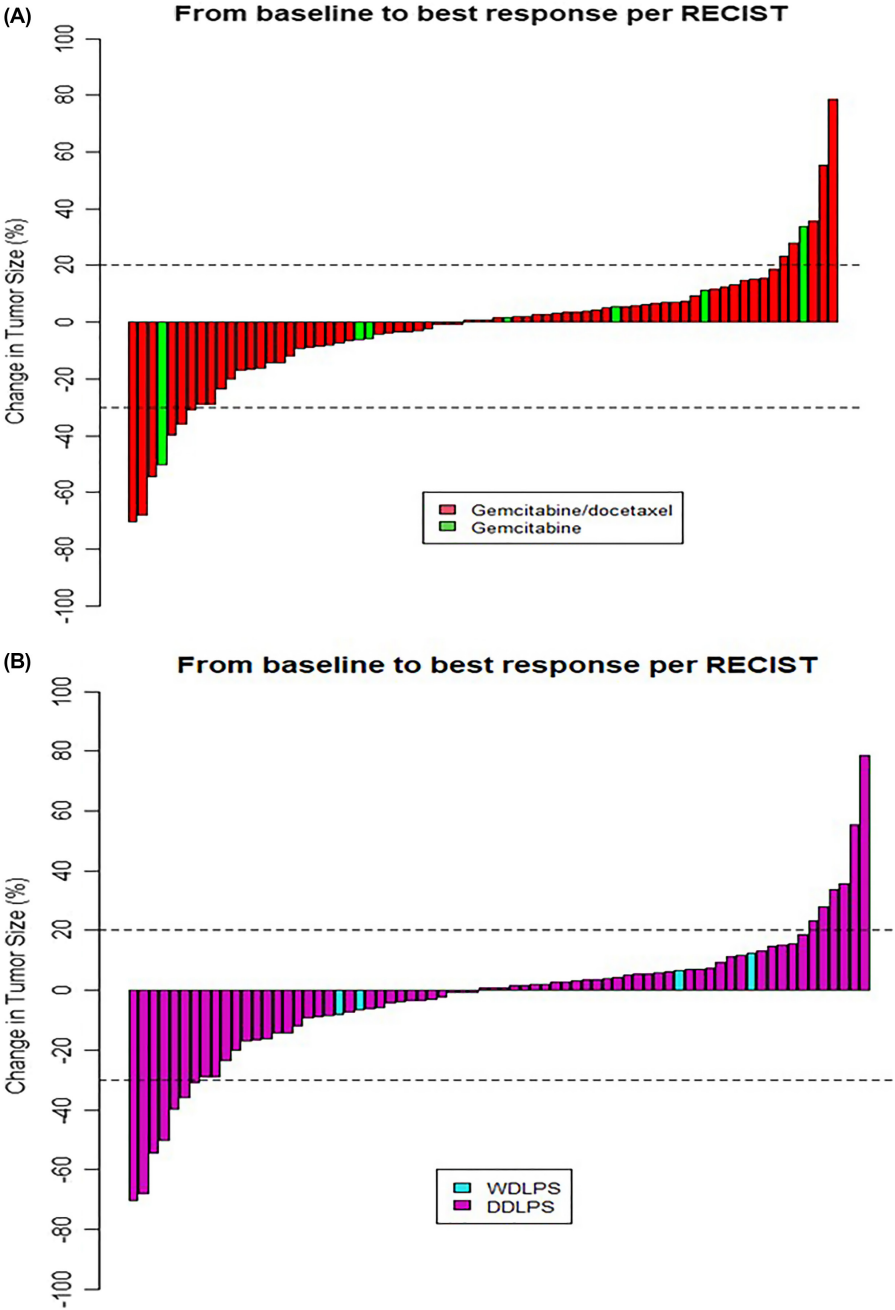
Waterfall plot of percentage change of tumor size. (A) Percentage change at best response, shown by regimen. (B) Percentage change at best response, shown by subtype at overall disease course.

Of all the 72 treatment instances of gemcitabine‐based chemotherapy, the RR, defined as the best response using RECIST 1.1 was 9.7% (7/72), the stable disease (SD) rate was 81.9% (59/72), and the progressive disease (PD) rate was 8.3% (6/72, Table S1A). All responses were observed only in those with documented DDLPS at treatment of gemcitabine‐based and at some point in their disease course (Figure 2). The RRs of gemcitabine‐docetaxel and gemcitabine as a single agent were 9.2% (6/65) and 14.3% (1/7), respectively. The SD rate at 3 months was 50.0% (36/72), including instances where the treatment was changed (13.9%, 10/72) prior to RECIST 1.1 progression. The 3‐month SD rate was 50.8% (33/65) for gemcitabine‐docetaxel and 42.9% (3/7) for gemcitabine as a single agent.

Patients underwent surgery after gemcitabine‐based treatment in 32 instances (44.4%, Table 2). Twenty‐one of these surgeries (65.6%) were immediately after the gemcitabine‐based treatment while 11 surgeries (34.4%) were after further intervening therapy (radiation and/or other chemotherapy regimens). From the 21 instances of surgery right after gemcitabine‐based treatment, 19 instances had an available surgical specimen reviewed by an independent sarcoma pathologist. Eleven of these 19 specimens showed tumor necrosis (57.9%). Among specimens with evidence of tumor necrosis, 8 specimens also had hyalinization representing necrosis due to the treatment (8/11, 72.7%). At least 30% necrosis was observed in 4 (4/19, 21.1%) of the surgical pathology specimens.

### 3.3 Survival outcomes

The median follow‐up time for TTP was 3.8 months (95% CI 3.0–4.5 months). A total of 31.9% instances (23/72) had confirmed PD by the end of this follow‐up time. The median TTP was 9.2 months (95% CI 5.3–12.3 months); the median TTP for gemcitabine‐docetaxel was 9.2 months (95% CI 5.3–12.5 months) while the median TTP for gemcitabine as a single agent was 6.8 months (95% CI 2.6 months–not reached). The log‐rank test did not show a significant difference in TTP between the 2 regimens (*p* = 0.676) or between settings (primary treatment/recurrent/metastasis; *p* = 0.775). The landmark analysis shows that TTP was not significantly different between the instances that achieved PR (*n* = 7) and those achieved SD (*n* = 59), with median TTPs of 5.4 months (95% CI 2.1 months–not reached) and 4.8 months (3.1 months, not reached) from time of best response, respectively (*p* = 0.82). Univariate analysis of TTP in relation to patient demographic and clinical factors showed no other factors of interest were significantly associated with TTP, including the type of regimen: gemcitabine as a single agent had a hazard ratio (HR) of 1.373 compared to gemcitabine‐docetaxel (*p* = 0.677; Table S2A). The analysis did reveal a trend of superior TTP with a gemcitabine dose of ≥900 mg/m^2^ (HR = 0.466, 95% CI 0.19–1.17, *p* = 0.10). Other factors, including line of treatment, setting, previous treatment with anthracycline, docetaxel dose used, and WDLPS versus DDLPS subtypes, did not show a significant influence on TTP.

For PFS analysis, 65 individual patients were included; to exclude multiple treatments in the same patient, PFS was calculated from the last gemcitabine‐based treatment initiation for the most recent recurrence. At the time of analysis, the median follow‐up for PFS was not reached (95% CI 19.1 months–not reached), and 45 of 65 (69.2%) patients had PD (as evaluated by RECIST 1.1) or death. The median PFS was 9.1 months (95% CI 6.7–10.5 months) for the total population, 9.2 months (95% CI 6.7–11.6 months) for the gemcitabine‐docetaxel group, and 6.8 months (95% CI 1.8–10.5 months) for the gemcitabine single‐agent group.

Since 7 of 65 patients (10.8%) in this study received gemcitabine‐based therapy more than once for separate recurrences, TTP and PFS were also analyzed with exclusion of patients who received the regimen more than once. In the remaining 58 patients, the median TTP was 9.2 months (95% CI 5.3–12.3 months) in the total cohort, 9.2 months (95% CI 5.3–12.3 months) in the gemcitabine‐docetaxel group (*n* = 54), and not reached (95% CI 2.6 months–not reached) in the gemcitabine single‐agent group (*n* = 4). The median PFS was 9.2 months (95% CI 6.7–11.6 months) in the total 58 patients, 9.2 months (95% CI 6.1–12.3 months) in the gemcitabine‐docetaxel group, and 10.5 months (95% CI 2.6–10.5 months) in the gemcitabine single‐agent group.

**FIGURE 3:**
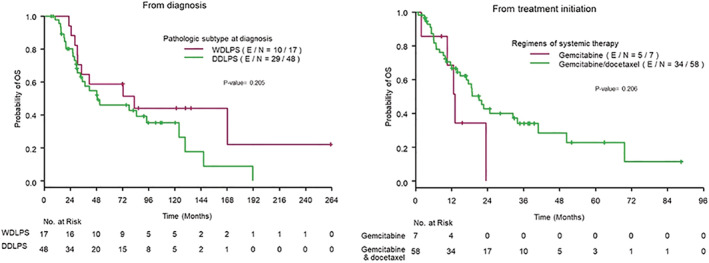
Kaplan–Meier curve of overall survival from diagnosis by subtype at diagnosis (left) and at treatment initiation (right).

Thirty‐nine of the 65 patients had died by the time of data cut‐off, with a median follow‐up of 102.5 months (95% CI 81.8–121.3 months). The median OS from diagnosis was 51.0 months (95% CI 34.1–123.9 months). The 5‐year OS rate was 50% (95% CI 36%–62%) while the 10‐year OS rate was 38% (95% CI 25%–51%). The median OS from diagnosis for those with DDLPS at initial diagnosis (*n* = 48) was 48.6 months (95% CI 30.4–123.9 months), and for those with only WDLPS at initial diagnosis (*n* = 17), the median OS was 82.6 months (95% CI 30.4 months–not reached). Of the 17 WDLPS patients, 13 developed DDLPS subsequently. The comparison of OS by subtypes at diagnosis showed a trend of better OS for WDLPS compared to DDLPS but did not reach statistical significance (*p* = 0.205, Figure 3). The median OS from the start of gemcitabine‐based therapy was 18.8 months (95% CI 13.1–32.4 months) in the total 65 patients, 21.1 months (95% CI 14.8–33.9 months) in the gemcitabine‐docetaxel group, and 13.1 months (95% CI 1.8–23.5 months) in the gemcitabine single‐agent group. There was no significant difference in OS from treatment initiation between regimens (*p* = 0.11).

Metastasis at diagnosis, surgery for primary disease, and number of surgeries were significantly associated with OS from diagnosis in the univariate setting (Table S3A). Metastasis at diagnosis and number of surgeries remained significant in the multivariate setting. Specifically, patients who had metastatic disease at diagnosis had inferior survival compared to those without metastatic disease at diagnosis (HR = 4.42, 95% CI 1.80–10.87, *p* = 0.0012), and those receiving more surgeries had superior survival compared to those receiving fewer surgeries (HR = 0.24, 95% CI 0.094–0.62, *p* = 0.003).

## 4 DISCUSSION

This study represents the largest database of gemcitabine‐docetaxel treatment outcomes in DDLPS. Although the efficacy of gemcitabine‐docetaxel has been reported in randomized studies of STS,^9^, ^10^ the heterogeneity among STS and even within different subtypes of LPS, precludes a true understanding of the efficacy in WDLPS/DDLPS.^17^ WDLPS is considered chemotherapy resistant, and DDLPS is known to have lower chemotherapy sensitivity compared to other STS subtypes; knowing the specifics of efficacy and outcome data for standard chemotherapy is important to help evaluate future novel options in this subtype. Of the 65 patients included in our analysis, 61 (93.9%) had histopathologically documented DDLPS during the course of their disease.

In STS patients, previous data from randomized studies suggest an RR per RECIST of 8% and 16%–20% in gemcitabine and gemcitabine‐docetaxel,^9^, ^10^ respectively. These studies had a limited number of LPS patients, with data on different LPS types not always reported. The percentage of each LPS subtype could substantially affect both the RR and survival outcomes from chemotherapy considering higher RR and PFS had been reported in myxoid/round cell LPS compared to DDLPS and pleomorphic LPS subtypes.^17^ While WDLPS demonstrated longer PFS than DDLPS and pleomorphic, despite being insensitive to chemotherapy due to its indolent biology. Efficacy of gemcitabine‐docetaxel in LPS was shown in a randomized controlled phase 2 of gemcitabine‐docetaxel with ontuxizumab. The gemcitabine‐docetaxel arm revealed median PFS of 24.1 weeks without further clarification available of the LPS subtype percentages.^18^ Also, a retrospective study in LPS from Korea showed an RR of 14.8%, although details on the percentage of LPS subtypes was not reported.^19^ Previously, our group reported on the efficacy of chemotherapy in WDLPS/DDLPS, mainly focusing on front‐line doxorubicin, and this study included a small number of patients who received gemcitabine‐docetaxel in the second line, in whom the RR was 17% (4/23).^20^ In the current study, which included a much larger number of WDLPS/DDLPS, with DDLPS being the majority of those studied; gemcitabine‐docetaxel had a RR of 9.72% (7/72), and all responses occurred in cases with DDLPS documented at the time of treatment. This RR appears to be lower than what one would expect for other STS subtypes, based on randomized studies, where RR ranged from 16%–20%.^9^, ^10^ A randomized study in non‐adipocytic STS also reported a higher RR of 18% (8/45) for gemcitabine‐docetaxel^21^ This outcome is likely due to differential chemotherapy sensitivity of DDLPS versus other STS, and also reflects the challenges of RECIST response assessment in this heterogeneous sarcoma subtype. DDLPS responds differently than WDLPS, with lower grade areas less likely to shrink, hence vascular/PET response criteria might be a better way to estimate response.^12^, ^13^ No significant difference in response was noted between gemcitabine‐docetaxel and gemcitabine as a single agent but given the very few patients receiving single‐agent therapy, one of whom had partial response, no definite conclusions can be drawn. In addition, TTP and OS did not show significant difference between the regimens with trend toward worse TTP and OS from treatment initiation in gemcitabine single agent subgroup (Figure 3).

Other potential second‐line regimens used in LPS include trabectedin and eribulin, based on randomized trials comparing their activity against that of dacarbazine in L‐type sarcomas (LPS and leiomyosarcoma).^22^, ^23^, ^24^, ^25^ In these randomized studies that led to the approval of these agents, L‐type sarcomas had an RR of 9.9% and 4% when treated with trabectedin and eribulin, respectively, but this number drops to 9% and 1.4%, respectively, in the LPS group. Of note, these studies also included other LPS subtypes such as myxoid/round cell and pleomorphic LPS, with the majority of the responses to trabectedin seen in the patients with myxoid/round cell LPS.

The clinical judgment by physicians of disease progression in LPS is often earlier than progression marked by RECIST 1.1. This difference is partly believed to be due to the selective evaluation of the DDLPS component and the combination of clinical symptoms of the patients. In the current study, the RECIST 1.1 response also did not correlate well with the response seen in the pathological specimens. Of the 19 specimens available from 21 patients who underwent surgery after gemcitabine‐based treatment, 4 specimens (21.1%) had ≥30% necrosis, and 8 specimens (42.1%) had both tumor necrosis of any percentage and hyalinization, which indicates a treatment effect. While tumor necrosis can happen from both the treatment effect and as a feature of tumor growth, hyalinization is believed to be more specific as a treatment effect. However, there is no validated method to assess pathological response in these sarcomas. Only a few publications have looked into the significance of tumor necrosis and hyalinization. One study showed that tumor necrosis of ≥90% is related to improved disease‐free survival on univariate analysis.^26^ In another study, hyalinization of >12.5% was related to relapse‐free survival and OS improvement.^27^ In our study, among the 8 tumor specimens with both necrosis and hyalinization, only 1 specimen was defined as PR by RECIST 1.1, with 5% necrosis and 15% hyalinization, while the rest were found to be SD. However, as detailed above, RECIST 1.1 is also imperfect measure of response in this subtype.

The median TTP and PFS per RECIST in our study were 9.2 months and 9.1 months, respectively, and compared favorably to the median PFS of 4.0–4.6 months, 3.0 months, and 2.9 months for doxorubicin‐based, trabectedin, and eribulin, respectively, for LPS.^20^, ^23^, ^24^, ^28^ Although, the presence of the 11.1% (8/72) WDLPS cases in our population might lead to longer TTP and PFS and would have been excluded in trabectedin and eribulin studies. The median PFS in STS has ranged from 5.9 to 6.2 months for gemcitabine‐docetaxel in randomized studies.^9^, ^10^ However, the TTP and PFS in our study as assessed by the treating physician were lower, at 4.3 and 3.8 months, respectively. Of the 33 instances (45.8%) of disease progression as evaluated by the treating physician, 23 (69.7%) did not have progression as evaluated by RECIST, as they did not reach 20% increase in sum of diameters of tumors from baseline, as required for PD.

The median OS from diagnosis in our study was 51.0 months (95% CI 34.1–123.9). The median OS from the initiation of gemcitabine‐based treatment was 18.8 months (95% CI 13.1–32.4), similar to what one would expect with a second‐line regimen in STS. In randomized studies of eribulin and trabectedin, the median OS was 15.6 months and 13.7 months, respectively, in LPS patients.^23^, ^24^, ^28^

Univariate and multivariate analyses identified 2 significant factors affecting OS of patients in our study (Table S3A; Figure S1A). Patients who underwent surgeries more than 2 times had a lower risk of death, with an HR of 0.242. This could be a consequence of the underlying biology of the disease and favorable patient factors; patients with good performance status and those with more limited disease are more amenable to surgery/debulking. Conversely, patients with metastatic disease at diagnosis had higher risk of death, with an HR of 4.42.

In conclusion, from our retrospective analysis, a gemcitabine‐based regimen is an efficacious second‐line treatment for DDLPS patients. The RR for this regimen was 9.7%, with some patients receiving prolonged benefit, and it compares favorably to later‐line options. Gemcitabine‐docetaxel can serve as a valid second‐line comparator arm in future clinical trials of DDLPS.

## AUTHOR CONTRIBUTIONS

**Prapassorn Thirasastr:** Data curation (lead); resources (lead); writing – original draft (lead); writing – review and editing (equal). **Heather Lin:** Formal analysis (lead); methodology (equal); software (lead); supervision (equal); validation (equal). **Behrang Amini:** Data curation (equal); investigation (equal); resources (equal). **Wei‐Lien Wang:** Data curation (equal); formal analysis (equal); investigation (equal); resources (equal); validation (equal); writing – review and editing (equal). **Jeffrey Cloutier:** Data curation (equal); investigation (equal); resources (equal). **Elise Nassif:** Methodology (equal); visualization (equal); writing – review and editing (equal). **Emily Z Keung:** Conceptualization (equal); supervision (equal); visualization (equal); writing – review and editing (equal). **Christina Roland:** Supervision (supporting); writing – review and editing (supporting). **Barry W Feig:** Supervision (supporting); writing – review and editing (supporting). **Dejka M. Araujo:** Visualization (supporting); writing – review and editing (supporting). **Robert S. Benjamin:** Supervision (supporting); writing – review and editing (supporting). **Anthony Paul Conley:** Supervision (supporting); writing – review and editing (supporting). **J Andrew Livingston:** Visualization (supporting); writing – review and editing (supporting). **Joseph A Ludwig:** Conceptualization (supporting); writing – review and editing (supporting). **Shreyaskumar R. Patel:** Supervision (supporting); writing – review and editing (supporting). **Ravin Ratan:** Visualization (supporting); writing – review and editing (supporting). **Vinod Ravi:** Visualization (supporting); writing – review and editing (supporting). **Maria Zarzour:** Supervision (supporting); writing – review and editing (supporting). **Xiao Zhou:** Project administration (lead); writing – original draft (supporting). **Neeta Somaiah:** Conceptualization (lead); supervision (lead); writing – original draft (equal); writing – review and editing (equal).

## FUNDING INFORMATION

None.

## CONFLICT OF INTEREST

Neeta Somaiah: Has a consulting or advisory role for Bayer, Deciphera, Boehringer Ingelheim, Aadi Biosciences, and Daiichi Sankyo; has received research funding (institutional) from MedImmune/AstraZeneca, GlaxoSmithKline, Karyopharm Therapeutics, Deciphera, Ascentage Pharma Group, Daiichi Sankyo, Eli Lilly.

## Supporting information


Tables S1A‐S3A

Figure S1A

## Data Availability

The data underlying this article will be shared on reasonable request to the corresponding author.
