# Supplementary material for: Retrospective evaluation of the role of gemcitabine‐docetaxel in well‐differentiated and dedifferentiated liposarcoma
Source: Cancer Med. 2022 Sep 24;12(4):4282–93. doi: 10.1002/cam4.5298 (PMC9972024; doi:10.1002/cam4.5298)
Supplement: Supplementary file 1 — Tables S1A‐S3A [file CAM4-12-4282-s001.docx]

**Supplementary Materials**

**Table 1A| Best response by RECIST 1.1 criteria**

| **Best response** | **Frequency (N=72)**  **(%)** | **Subtype*** | | **Regimen** | |
| --- | --- | --- | --- | --- | --- |
|  |  | **WDLPS (n=8)**  **(%)** | **DDLPS (n=64)**  **(%)** | **Gemcitabine-docetaxel (n=65)** | **Gemcitabine single agent (n=7)** |
| Partial response | 7  (9.7%) | 0  (0%) | 7  (10.9%) | 6  (9.2%) | 1  (14.3%) |
| Progression | 6  (8.3%) | 0  (0%) | 6  (9.4%) | 5  (7.7%) | 1  (14.3%) |
| Stable disease | 59  (81.9%) | 8  (100%) | 51  (79.7%) | 54  (83.1%) | 5  (71.4%) |
| Stable at 3 months  NA due to regimen change before PD  NA due to local therapy before 3 months  NA due to no imaging at 3 months | 36  (50.0%)  10  (13.9%)  9  (12.5%)  6  (8.3%) | 6  (75.0%)  0  (0%)  1  (12.5%)  1  (12.5%) | 30  (46.9%)  10  (15.6%)  8  (12.5%)  5  (7.8%) | 33  (50.8%)  9  (13.8%)  8  (12.3%)  5  (7.7%) | 3  (42.9%)  1  (14.3%)  1  (14.3%)  1  (14.3%) |

NA, not assessed; PD, progressive disease; WDLPS, well-differentiated liposarcoma; DDLPS, dedifferentiated liposarcoma

*Pathologic subtype at the time of gemcitabine-based treatment

**Table 2A | Univariate analysis of time to progression per RECIST in relation to demographic and clinical factors**

| **Parameter** | **Univariate analysis** | | |
| --- | --- | --- | --- |
|  | **HR** | **95% CI** | **p-value** |
| Lines of treatment ≥3 | 0.965 | 0.32-2.94 | 0.95 |
| Metastatic status | 1.339 | 0.55-3.24 | 0.52 |
| Surgeries ≥1 | 1.873 | 0.78-4.49 | 0.16 |
| Presence of DDLPS* | 1.650 | 0.48-5.70 | 0.43 |
| Previous treatment with anthracycline | 1.194 | 0.49-2.91 | 0.70 |
| Gemcitabine ≥900 mg | 0.466 | 0.19-1.17 | 0.10 |
| Docetaxel >60 mg/m^2^/cycle | 1.396 | 0.49-3.95 | 0.53 |
| Regimen (gemcitabine single agent vs gem-tax) | 1.373 | 0.31-6.11 | 0.68 |
| Male gender | 0.721 | 0.31-1.68 | 0.45 |
| Presence of DDLPS at diagnosis | 1.200 | 0.49-2.96 | 0.69 |
| Presence of DDLPS at any point | 1.500 | 0.34-6.60 | 0.59 |
| Presence of multifocal disease | 0.894 | 0.23-3.47 | 0.87 |
| Setting (Recurrent/metastatic) | 1.657 | 0.76-3.60 | 0.20 |
| Best response (reference: PR)   - SD | 1.14 | 0.36-3.6 | 0.82 |

DDLPS, dedifferentiated liposarcoma; HR, hazard ratio; PR, partial response; SD, stable disease; PD, progressive disease

*Presence of evidence of DDLPS subtype before treatment

**Table 3A |** **Univariate and multivariate analysis of overall survival in relation to demographic and clinical factors**

| **Parameter** | **Univariate analysis** | | | **Multivariate analysis** | | |
| --- | --- | --- | --- | --- | --- | --- |
|  | **HR** | **95% CI** | **p-value** | **HR** | **95% CI** | **p-value** |
| Age at diagnosis | 1.019 | 0.99-1.05 |  | - | - | - |
| Male gender | 1.298 | 0.68-2.50 | 0.435 | - | - | - |
| Lines of treatment ≥3 | 0.509 | 0.25-1.02 | 0.057 | - | - | - |
| Surgeries ≥3 | 0.329 | 0.14-0.80 | 0.015 | 0.242 | 0.09-0.62 | 0.0030 |
| Presence of DDLPS at diagnosis | 1.595 | 0.77-3.31 | 0.209 | - | - | - |
| Presence of DDLPS at any point | 0.792 | 0.28-2.28 | 0.665 | - | - | - |
| Presence of metastatic disease at diagnosis | 2.733 | 1.19-6.29 | 0.018 | 4.421 | 1.80-10.87 | 0.0012 |
| Presence of metastasis at any point | 1.263 | 0.61-2.60 | 0.528 | - | - | - |
| Primary location at retroperitoneum | 0.948 | 0.49-1.83 | 0.873 | - | - | - |
| Surgery as primary treatment | 0.074 | 0.02-0.29 | 0.0002 | - | - | - |

DDLPS, dedifferentiated liposarcoma; HR, hazard ratio
