# Supplementary figures and images for: Retrospective evaluation of the role of gemcitabine‐docetaxel in well‐differentiated and dedifferentiated liposarcoma
Source: Cancer Med. 2022 Sep 24;12(4):4282–93. doi: 10.1002/cam4.5298 (PMC9972024; doi:10.1002/cam4.5298)

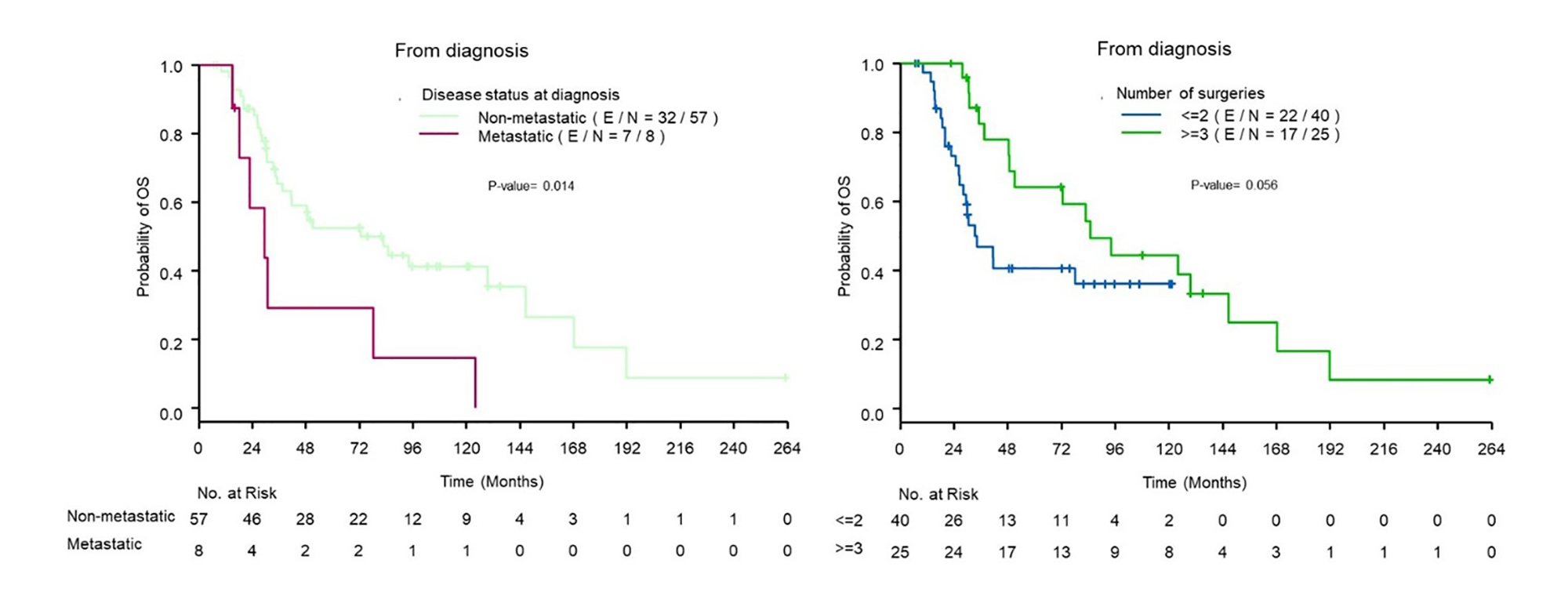

Supplement: Supplementary file 2 — Figure S1A [file CAM4-12-4282-s002.jpg]
